# Improving early childhood care and development, HIV-testing, treatment and support, and nutrition in Mokhotlong, Lesotho: study protocol for a cluster randomized controlled trial

**DOI:** 10.1186/s13063-016-1658-9

**Published:** 2016-11-09

**Authors:** Mark Tomlinson, Sarah Skeen, Marguerite Marlow, Lucie Cluver, Peter Cooper, Lynne Murray, Shoeshoe Mofokeng, Nathene Morley, Moroesi Makhetha, Sarah Gordon, Tonya Esterhuizen, Lorraine Sherr

**Affiliations:** 1 Department of Psychology, Stellenbosch University, Stellenbosch, South Africa; 2 Department of Social Policy and Intervention, Oxford University, Oxford, UK; 3 Department of Psychiatry and Mental Health, University of Cape Town, Cape Town, South Africa; 4 University of Reading, Reading, UK; 5 Leadership Management and Governance Project, Management Sciences for Health, Pretoria, South Africa; 6 Centre for Evidence-Based Health Care, Stellenbosch University, Stellenbosch, South Africa; 7 University College London, London, UK

**Keywords:** Early childhood development, HIV, Nutrition, Lesotho, Maternal and child health, Community health workers, Paraprofessionals

## Abstract

**Background:**

Since 1990, the lives of 48 million children under the age of 5 years have been saved because of increased investments in reducing child mortality. However, despite these unprecedented gains, 250 million children younger than 5 years in low- and middle-income countries (LMIC) cannot meet their developmental potential due to poverty, poor health and nutrition, and lack of necessary stimulation and care. Lesotho has high levels of poverty, HIV, and malnutrition, all of which affect child development outcomes. There is a unique opportunity to address these complex issues through the widespread network of informal preschools in rural villages in the country, which provide a setting for inclusive, integrated Early Childhood Care and Development (ECCD) and HIV and nutrition interventions.

**Methods:**

We are conducting a cluster randomised controlled trial in Mokhotlong district, Lesotho, to evaluate a newly developed community-based intervention program to integrate HIV-testing and treatment services, ECCD, and nutrition education for caregivers with children aged 1–5 years living in rural villages. Caregivers and their children are randomly assigned by village to intervention or control condition. We select, train, and supervise community health workers recruited to implement the intervention, which consists of nine group-based sessions with caregivers and children over 12 weeks (eight weekly sessions, and a ninth top-up session 1 month later), followed by a locally hosted community health outreach day event. Group-based sessions focus on using early dialogic book-sharing to promote cognitive development and caregiver-child interaction, health-related messages, including motivation for HIV-testing and treatment uptake for young children, and locally appropriate nutrition education. All children aged 1–5 years and their primary caregivers living in study villages are eligible for participation. Caregivers and their children will be interviewed and assessed at baseline, after completion of the intervention, and 12 months post intervention.

**Discussion:**

This study provides a unique opportunity to assess the potential of an integrated early childhood development intervention to prevent or mitigate developmental delays in children living in a context of extreme poverty and high HIV rates in rural Lesotho. This paper presents the intervention content and research protocol for the study.

**Trial registration:**

The Mphatlalatsane: Early Morning Star trial is registered on the International Standard Randomized Controlled Trial Number database, registration number ISRCTN16654287; the trial was registered on 3 July 2015.

## Background

Since 1990, the lives of 48 million children under the age of 5 years have been saved because of increased investments in reducing child mortality [1]. However, despite these unprecedented gains, 250 million children younger than 5 years in low- and middle-income countries (LMIC) fail to meet their developmental potential because they are living in contexts of poverty, poor health and nutrition, and without necessary stimulation and care [2]. Infants and young children develop best when caring adults respond with warmth and consistency and when they are provided with opportunities for interaction and learning [3, 4]. However, when caregivers are living in circumstances characterized by extreme poverty, poor nutrition and a high burden of disease, such as HIV/AIDS, their capacity to fulfill the caregiving role is compromised, placing the child at high risk for poor cognitive and socioemotional development [5].

Lesotho is a country with high levels of poverty, where 40 % of the population lives below the international poverty line of US$1.25 a day and 77 % of the population lives in rural areas [6, 7]. Most of the country is mountainous, and many villages remain accessible only by foot or on horseback. It has one of the highest adult HIV prevalence rates globally, at 23 %, and is home to an estimated 36,000 children living with HIV [7] as well as large numbers of children orphaned by HIV/AIDS [8]. Early diagnosis of HIV infection is essential to promote child survival and development, but geography and severe climate present challenges to HIV-testing and treatment of children. Difficult terrain and long periods of snow cover contribute to low rates of testing and long delays in obtaining infants’ test results [9] and antiretroviral (ART) coverage among children aged 0–14 years is only 25 %, the lowest in southern Africa [10]. In recent years, Lesotho has made significant progress in the rollout of HIV-testing and treatment for adults, but has not reached national testing treatment targets, and increasing testing rates and expanding treatment coverage remains a major challenge [11].

The potential effect of the high HIV rate and poor treatment coverage on children in the country is enormous. In addition to the risk to their survival, children affected by HIV/AIDS are at serious risk of developmental delays. HIV affects child development both directly and indirectly; there is evidence of cognitive delays for children who are HIV-infected, and also for those exposed to HIV in utero but born uninfected, and for those whose parents are affected by HIV [12].

In addition, due to the climate and mountainous terrain, and resulting poor food security, an estimated 13 % of children in the country are moderately or severely underweight, and 39 % have moderate or severe stunting [8].

Delivering interventions in the early years has been shown to be cost-effective [13], to reduce inequities [14] and to substantially improve long-term adult health outcomes [15]. Sustainable gains in child survival and development have been generated by social interventions and health care initiatives that seek to improve the physical and mental health of caregivers, support child nutrition, secure basic good-quality health services, and build culturally compatible linkages between service programs and communities [16, 17].

To date, however, maternal and child health intervention strategies have tended to address only one health risk at a time [18] and the practice of combining health, nutrition, and Early Childhood Care and Development (ECCD) interventions for their additive impact has been limited [19–21]. There is a unique opportunity to promote child development outcomes in Lesotho through the widespread network of informal preschools in rural villages in the country. Daycare centers, crèches, community-based childcare, or formal preschool services offer many advantages for communities affected by HIV/AIDS. ECCD centers can act as an entry point providing multiple health and social services, including facilitating access to HIV treatment and care [22].

## Methods

### Objectives

We are conducting a cluster randomized controlled trial to evaluate an intervention called “Mphatlalatsane: Early Morning Star,” which integrates ECCD, HIV-testing and treatment services, and nutrition education, for caregivers with children aged 1–5 years in informal preschool settings in rural communities near Mokhotlong, Lesotho. Data are collected at baseline prior to the intervention, post intervention, and at 12 months post intervention.

We hypothesize that, compared to participants in control villages, caregivers and their children in the intervention villages will have:

- Higher rates of HIV-testing for children
- Better child cognitive and language outcomes
- Increased uptake of ART for HIV+ children after testing
- Higher rate of adherence to ART for HIV+ children
- Better child growth status

### Collaboration

“Mphatlalatsane: Early Morning Star” is a collaborative project run by Stellenbosch University, University College London, the University of Oxford, and the University of Reading, in collaboration with the Management Sciences for Health Leadership Management and Governance Project, GROW, a local nongovernmental organization which is active in the Mokhotlong area, and the Ministry of Education and Training of Lesotho. It is funded by PEPFAR’s Orphans and Vulnerable Children Special Initiative, a series of three research projects testing integrated early interventions in southern African countries. Funders do not have direct involvement in study design or management, but may use published papers to write reports after data analysis and reporting is complete. In addition, data may be used in joint publications with data from the two other projects in the Special Initiative Program.

The “Mphatlalatsane: Early Morning Star” project has a Community Advisory Board that meets quarterly, consisting of a number of local key stakeholders, and a Data Safety and Monitoring Board, made up of international experts, that meets biannually. This is an independent group of people without any competing interests.

### Research ethics and approval

The study has research ethics approval from the Health Research Ethics Committee at Stellenbosch University, reference number N14/09/127 and the Lesotho Ministry of Health Ethics Committee, reference number 138-2014.

If there are any indications of negative effects, as noted in process observations or reports to the Research Ethics Committees, Community Advisory Board, or Data Safety and Monitoring Board, the principal investigators and partners will be alerted, and preventative action taken.

### Study setting

**Fig. 1:**
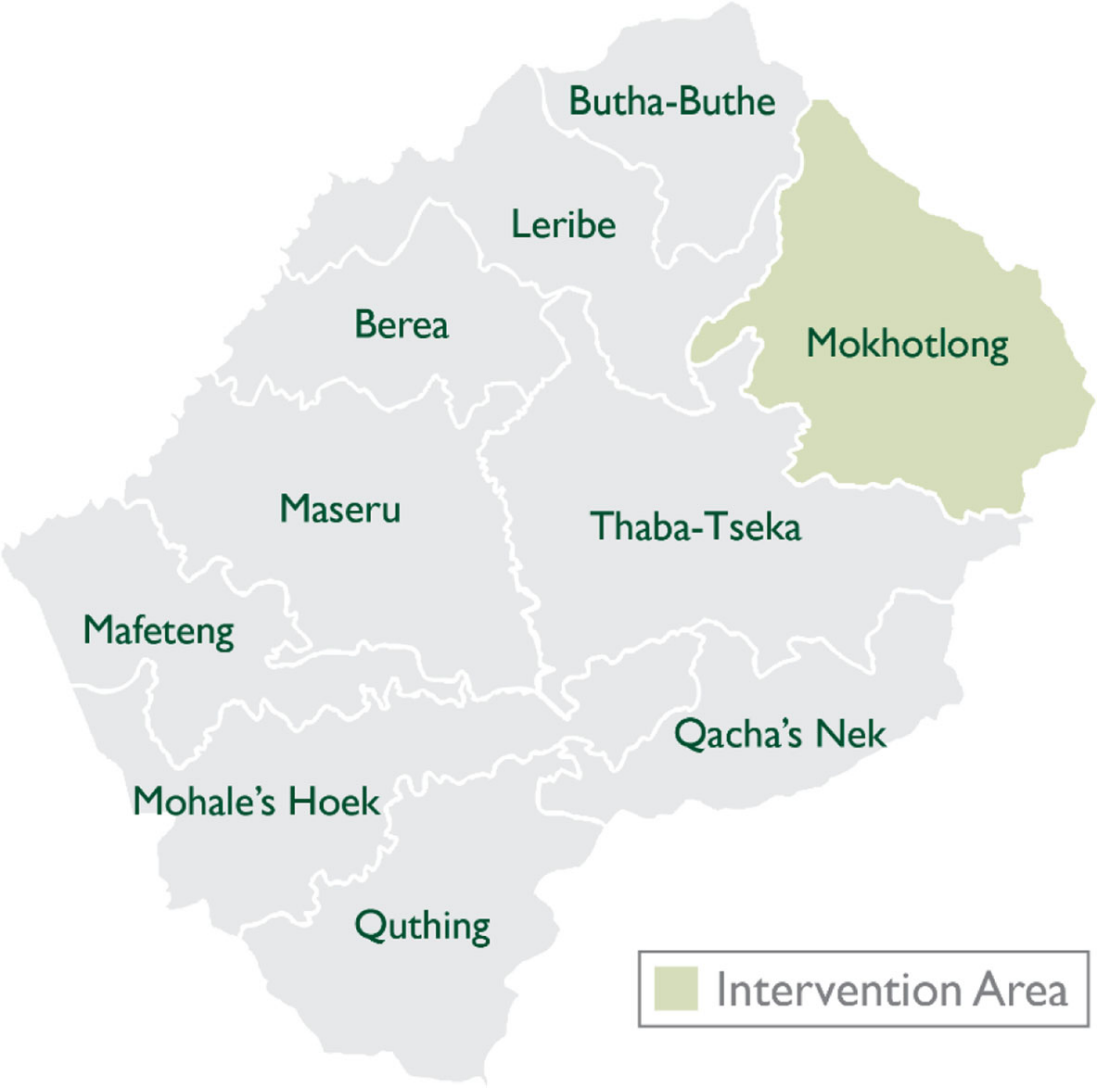
Location of Mokhotlong district, Lesotho

The study takes place in 34 villages in the Mokhotlong district in north-eastern Lesotho (see Fig. 1). Villages are very remote, with poor transport and road facilities, and severe weather conditions, especially in the winter months. The intervention is delivered in informal preschools in rural villages in the district. All children in the villages are included, regardless of whether or not they regularly attend the preschool. These informal preschools are run by local community members, often in their own homes, which are usually stone and mud houses without electricity or running water. Preschool staff have limited or no toys, books, or equipment, but are usually provided with support from the district Ministry of Education and Training in the form of monthly meetings and training workshops.

### Neighborhood selection, matching and randomization

The selected clusters for the study were identified as villages where GROW had a resident community volunteer, and where there were informal preschool centers in operation. It was important to identify villages with an operational ECCD center, since the study makes use of these facilities to deliver the intervention to young children and their primary caregivers.

Prior to cluster randomization, all eligible villages were mapped in order to determine the approximate number of children aged 1–5 years per village, the characteristics of the communities, the number of preschool centers, and the available government and nongovernmental services. Mapping (and subsequent data collection) in some villages requires transport by donkey due to inaccessibility by vehicle. Trained field-workers conducted the mapping exercise in two stages. During the primary mapping exercise, field-workers collected information at village level, preschool center level, and health care level for all villages. During the secondary mapping exercise, field-workers returned to the two largest, the two mid-size, and the two smallest villages to identify the number of children aged 1–5 years in each household.

Based on the village mapping activity, 34 villages were matched into pairs based on various community, preschool and health service characteristics, and stratified by size and relative remoteness. In each pair, villages were then randomly assigned to the intervention group, where participants are allocated to the intervention or the control group using a free web-based randomization program [23]. Randomization was overseen by an external statistician and the procedure followed Consolidated Standards of Reporting Trials (CONSORT) guidelines [24]. The overall sample of villages was divided into two phases: phase 1 and phase 2, with phase 1 IC villages receiving the intervention first, followed by the roll-out of the intervention in phase 2 IC villages.

Researchers notified intervention and control village leadership, preschool staff, and community members about their allocation individually. All data collectors and data coders are blinded to group allocation in order to minimize assessment bias [23] and study participants are asked not to reveal their condition to data collectors at any point.

### Intervention

The intervention is a group-based intervention which consists of three components: (1) “Book-sharing,” (2) “Health” (specific focus on HIV-testing and treatment), and (3) “Growth” (nutrition education) in addition to a Community Health Outreach Day at the end of the intervention period.

The ECCD component consists of caregiver training in sensitive “book-sharing” skills, designed to stimulates the child cognitively and encourage caregiver-child engagement [25, 26]. The benefits of dialogic book-sharing have been well-established in high-income countries and include language development, attention, literacy, and school readiness [27]. In LMIC, there is limited evidence available, but a previous study in South Africa found that a book-sharing intervention for caregivers of 14–16 month-old infants living in an impoverished periurban settlement proved effective in improving children’s language development, sustained children’s attention as well as socioemotional development, and led to improvements in carer-child interaction, increasing carers’ sensitivity to their infant’s interests and cues [25–27]. In this project, the book-sharing intervention program principles have been combined with a participatory, evidence-based approach to addressing issues around HIV-testing and nutrition education, based on local needs and resources.

#### Selecting intervention facilitators and community-based mentors

The intervention is delivered by trained and supervised community health workers, with an intervention facilitator and a community-based mentor in each team. The intervention facilitator delivers the book-sharing component of the intervention while the community-based mentor delivers the HIV and nutrition components. Intervention facilitators and community-based mentors were selected in a two-stage process. Firstly, 30 applicants were selected to attend an interview based on their experience and/or prior training in working with children and facilitation of group activities. Each potential candidate attended an interview with a panel of interviewers from GROW and Stellenbosch University. Of these, we selected 26 candidates to attend a 7-day training workshop. This workshop was used to help the panel to select the final team of one intervention coordinator, eight intervention facilitators, and eight mentors.

#### Training

Training took place over several phases. First, we conducted the preselection training workshop, which covered basic training in book-sharing and facilitation skills. Next, candidates selected as intervention facilitators and community-based mentors attended a pilot intervention training workshop over 5 days. This training session covered informed consent procedures, referral mechanisms, the use of tablet devices for the intervention delivery, and troubleshooting for intervention sessions. Facilitators and mentors were primarily trained separately on their respective session content, but both attended selected book-sharing and health and nutrition training sessions in order to support their partners and become familiar with the integrated nature of intervention delivery. The training also included monitoring and evaluation procedures such as scheduling sessions, documenting attendance, making referrals, and completing feedback questionnaires after every session.

Each training workshop followed a similar structure and approach to participant training. Didactic sessions were followed by opportunities for practice. During practice sessions, the training facilitators offered frequent positive feedback and support. Trainers used tokens, goal-setting, and an emphasis on positive feedback to establish a warm and supportive group environment that encouraged staff members to fully participate and feel empowered in their roles as intervention facilitators and community-based mentors. It was important to model this approach to training participants as this is the same approach that they in turn are expected to incorporate in their delivery of the intervention sessions.

#### Supervision

Intervention supervision takes place in the form of weekly group supervision meetings during the intervention delivery period to discuss challenges and successes from the past week. This includes difficult situations that may have arisen in the group setting, practical issues, and issues affecting fidelity such as maintaining good attendance levels. In addition, a supervisor conducts site visits on an ad hoc basis during the 8-week period, documents performance, and provides feedback. Each team is visited between three and four (every second week) times over the 8-week period. In addition, intervention teams are encouraged to report on a daily basis, via a Whatsapp group chat, on general progress, challenges, referrals, and any other concerns in the field.

#### Pilot

The Medical Research Council framework recommends a feasibility and piloting phase after an intervention has been developed [28]. Because this is a newly developed integrated program, two short intervention pilots were conducted in order to assess the feasibility and acceptability of the program, to assess logistical issues and to test the intervention content. The pilots were conducted over a period of 4–5 weeks in nine preschools in Mokhotlong town. During this period GROW and Stellenbosch University, with technical support from Management Sciences for Health, piloted key components of the intervention program and some research procedures. After this pilot period, focus groups were conducted with participants about their experiences of the intervention, as well as with other local stakeholders/experts. The final intervention materials were then refined to reflect local contextual issues, such as geographic challenges and family composition.

#### Intervention protocol

The intervention consists of group sessions with five to six caregivers and their children which last for 2 to 3 h, and are held weekly for eight consecutive weeks. Following the eighth session, after 1 month, a ninth “top up” session takes place, after which a community health outreach day is held in a location accessible to the village. The number of groups held in each village varies according to the size of the village. For 12 months thereafter, selected, appropriate books for young children are dropped off in intervention villages on a monthly basis to encourage groups to continue to meet regularly.

#### Group sessions

The group intervention content is specified in a manual which is used to train intervention staff. The intervention consists of three components: (1) “Book-Sharing” (Early Childhood Care and Development), (2) “Health” (specific focus on HIV-testing and treatment), and (3) “Growth” (nutrition education). The intervention session content consists of specific tasks for each session (see Table 1 for a description of intervention content). Children are divided into groups based on their age so that younger children (aged 12–30 months) and older children (aged 31–60 months) receive the intervention in different groups, and the intervention content differs slightly according to age groups.

**Table 1:** Intervention session content

	Nutrition and health (all ages)	ECCD for children aged 12–30 months	ECCD for children aged 31–60 months
Session 1	Introduce the importance of child health and growth for future development	Introduce concept of picture book-sharing as an activity that improves caregiver-child relationship and facilitates school readiness	Introduce concept of picture book-sharing as an activity that improves caregiver-child relationship and facilitates school readiness
	Present child development as a holistic concept (physical, mental and social health)	Facilitate baby’s handling of the book	Encourage child’s active participation in book-sharing
Session 2	Importance of growth monitoring	Support caregivers to follow cues from baby	Encourage pointing and naming to help child learn new words
	Basic nutrition education	Encourage baby’s active participation in book-sharing	Engaging the child in a conversation about the picture book (using “where,” “who,” “what,” “why” questions)
	Identifying locally available nutritious food options	Demonstrate how to follow baby’s interest (indexed by simple looking, patting or banging the picture)	Active linking of book content to the child’s world
	Measuring weight, height and MUAC	Using lively voice to keep baby interested and to help baby learn	Responding to the child in a supportive and nonjudgmental manner
	Individual feedback session with caregivers to discuss child’s growth status and make referrals if necessary		
Session 3	Key messages and recommendations for appropriate infant and young child feeding	Using pointing and naming of pictures to help baby learn new words	Talking about feelings with the child: using book-sharing as an opportunity to talk about different emotions
	Hygiene, sanitation, and safe food preparation	Repeating words to help baby remember new words	Helping child understand the meaning of emotion words
	Early recognition of illness and help-seeking	Emphasizing the importance of positive responses	Linking emotions being shown in the book to the child’s own emotions
		Finding opportunities to praise baby	
Session 4	Information about HIV prevention and modes of HIV infection	Prompting baby to point to words that he/she knows (“where is the…” or “can you find the…”)	Talking about intentions: helping child to think about reasons why characters in the book are doing what they are doing (questions such as “why do you think they are doing that?” or “what are they trying to do here?”)
	Importance of early identification of HIV (video followed by group discussion)	Prompting baby to name objects in the book that he/she knows how to say by asking “what’s this?”	
Session 5	Emphasizing the importance of early identification of HIV through testing for caregivers and children	Active linking of book content to the baby’s world (making links between objects in the book and objects in the baby’s visual field)	Perspective taking: using what is happening in the book to help the child see that different people can think and feel different things
	Information on benefits of HIV treatment (video followed by group discussion)		
Session 6	Exploring fears and concerns (for family testing)	Talking about feelings with the child: using book-sharing as an opportunity to talk about different emotions	Numeracy and comparisons: using the book to help child become comfortable with numbers and practice counting
	Discuss barriers to testing and disclosure	Helping child understand the meaning of emotion words	Using books to get children to make comparisons in relation to numbers, size or order (most/least; big/small; first/last)
	Identify strategies and systems of support to overcome barriers	Linking emotions being shown in the book to the child’s own emotions	
Session 7	Summary of key messages from past 6 sessions	Summary of main book-sharing principles from past 6 sessions using video examples of caregivers	Summary of main book-sharing principles from past 6 sessions using video examples of caregivers
Sessions 8 & 9	Promote attendance of community health events	Review of main principles using more video examples of caregivers	Review of main principles using more video examples of caregivers
	Identify strategies for continued recognition of illness and help-seeking	Identify strategies to encourage continued book-sharing	Identify strategies to encourage continued book-sharing

*ECCD* Early Childhood Care and Development, *MUAC* mid upper arm circumference

The intervention is hosted in local village preschools. The intervention facilitators and community-based mentors work together to deliver the sessions, each focusing on a different component of the intervention. Each intervention session consists of a group session, followed by short individual sessions. The sessions include group and individual activities and make use of presentations shown on a tablet computer as well as practical exercises. The aim is to convey didactic information, to model key skills, and to facilitate and encourage caregiver in dialogic book-sharing [25]. This is achieved using a group presentation together with video material. Following the presentation, each caregiver-child pair has a brief period of sharing a picture book together during which the facilitator makes suggestions and provides support. The Health and Growth components consist of key educational messages and facilitating the identification of available resources/services in the primary caregiver’s environment to motivate positive practices. Community-based mentors conduct activities with caregivers, present visual aids, facilitate group discussions and practical demonstrations to convey important messages for nutrition and the importance of testing for HIV. They are responsible for making referrals and provide follow-up support to children who test positive for HIV during the intervention.

#### Community health outreach days

After the ninth intervention session, local organizations are mobilized to coordinate accessible community health events that aim to increase HIV-testing among families through promoting an overall focus on child health. They take place in several locations over a 4-week period and are targeted at both intervention and control villages. The events are structured in clusters of communities based on their geography. The community health outreach days provide a wide range of services which include nutrition assessments, vaccinations, HIV-testing and counseling, general health consultations, birth document registration, and delivery of information on child protection and community gardening. Project partners work with a number of local partners to run these events: Baylor College of Medicine Children’s Foundation Lesotho, the Child and Gender Protection Unit, the Ministry of Health’s District Health Management Team, the Food and Nutrition Coordinating Office, the Ministry of Agriculture and Food Security, the National Identity Civil Registry, and a local nongovernmental organization called Touching Tiny Lives.

### Control condition

The control villages in the study do not receive the group-based integrated intervention, but they are invited to take part in community health outreach days that take place in an accessible venue near to their homes and provide access to a range of services, including HIV-testing and counseling and nutrition assessments. They have access to standard health services available in Lesotho, primarily delivered through primary health care services in the district. In addition, each control village has an informal paid preschool service available in their village.

### Participant recruitment

All children aged 1–5 years at the time of baseline assessment who are living in study villages are eligible for participation in the study, regardless of whether or not they attend the paid village preschool. They are enrolled together with their primary caregivers. Adult primary caregivers enrolled in the study are included if they are 18 years or older, serve as a primary caregiver for the child, live in the same house as the child at least four nights per week, and provide consent for themselves and their child to participate in the study including intervention and completing baseline, and immediate post test and 12-month follow-up assessments.

After agreement from the local chief and community leadership, trained recruiters go door-to-door in villages to identify children within the age range of the study. They collect basic demographic information on participants who agree in principle to take part. At a later stage, following a recruitment visit at which formal consent to participate in the trial is obtained, there is a further visit from a baseline data collector.

### Data collection

#### Data collector training

Data collector training consists of principles of research ethics, referral procedures, and management of difficult situations in the field. The data collector team members have learned how to complete the informed consent procedure with a focus on how to handle participants with low literacy levels, and how to administer the questionnaire including dealing with sensitive issues. In addition team members have been extensively trained on how to administer the child developmental assessments that are used in the study. This includes principles of child assessment, how to set up the assessment space, establishing rapport with young children, child seating and positioning, and how to minimize and manage distractions in the community setting.

#### Procedures

Table 2 shows the study schedule which follows the Standard Protocol Items: Recommendations for Interventional Trials (SPIRIT) guidelines.

**Table 2:** Schedule of enrollment, interventions, and assessments following the Standard Protocol Items: Recommendations for Interventional Trials (SPIRIT) figure

	Allocation	Enrollment	Post allocation					Close-out
			Weeks 1–8	Weeks 6–18	Weeks 16–24	Weeks 21–25	Weeks 68–76	
Time point	0		Baseline	Group intervention	Post-intervention follow-up	Community health outreach day	12-month follow-up	18 months post intervention
Enrollment:		X	X					
Eligibility screen		X						
Informed consent			X	X	X		X	
Allocation	X							
Interventions:								
Intervention condition				X		X		
Control condition						X		
Assessments:								
Primary outcomes:								
HIV-testing rate			X		X		X	
Child language			X		X		X	
Child attention			X		X		X	
Secondary outcomes:			X		X		X	
Child cognitive development			X		X		X	
Child growth			X		X		X	
HIV treatment uptake			X		X		X	
HIV treatment adherence			X		X		X	
Child emotional and behavioral functioning			X		X		X	
Child executive functioning			X		X		X	
Parental discipline			X		X		X	
Parenting stress			X		X		X	
Parental sensitivity			X		X		X	
Caregiver mental health			X		X		X	
Alcohol use			X		X		X	
Mediators:			X		X		X	
Positive parenting			X		X		X	
Stimulation of child at home and preschool			X		X		X	
Moderators:								
Socioeconomic status			X		X		X	
Family structure/caregiver relationship			X		X		X	
Caregiver mental health			X		X		X	
Child gender		X						
Quality/fidelity of intervention received			X		X		X	

The study flowchart is included in Table 3. Data are collected at baseline and again at two follow-up points: 2 weeks post intervention and 12 months post intervention. Data collectors are blind to intervention group. They travel to the villages in teams and remain in each village over several days while data collection is underway. They first meet with potential participants and conduct the informed consent process before starting any research activities. All data collection takes place in rented houses within the villages which provide a quiet and private space for all interviews and assessments. Following data collection participants are provided with a small package of groceries for their time, consisting of soap, washing powder, breakfast bars, and juices.

**Table 3:** Primary and secondary outcome measures

Outcome	Measure
Primary outcomes	
HIV-testing rate	Number of children who have tested for HIV in the past 3 months
Child language	MacArthur Communication Development Inventory (CDI)
	Peabody Picture Vocabulary Test (PPVT)
	Mullen Scales of Early Learning: Receptive Language Scale
Child attention	Early Childhood Vigilance Task (ECVT)
Secondary outcomes	
Child cognitive development	Mullen Scales of Early Learning: Visual Reception Scale
Child growth	*Z*-scores, based on WHO standards
HIV treatment uptake	Number of children initiating ART therapy after testing
HIV treatment adherence	ART adherence rates in children, defined as % adhering to ART within defined periods (3 days, 1 week, 1 month)
Child emotional and behavioral functioning	Child Behavior Checklist (selected subscales)
	Strengths and Difficulties Questionnaire
Child executive function	Inhibitory control task
	Attention shifting task
	Working memory task
Parental discipline	Discipline and Violence Questionnaire of Lansford and Deater-Deckard, developed from the Parent-Child Conflict Tactics Scale and the World-SAFE survey
Parenting stress	Parenting Stress Index (PSI)
Parental sensitivity	Directly observed book-sharing task rated with adapted version of the Murray Global Rating Scales
	Directly observed problem-solving task rated with adapted version of the Murray Global Rating Scales
Caregiver mental health	Patient Health Questionnaire-9 (PHQ-9)
	Generalized Anxiety Disorder-7 (GAD-7)
	Self-Reporting Questionnaire-20 (SRQ-20)
	Shona Symptom Questionnaire
Caregiver alcohol use	Alcohol Use Disorders Identification Test (AUDIT)

*ART* antiretroviral therapy, *WHO* World Health Organization

Data are collected by the data collectors on tablet computers, which instantly transmit participant responses to a server, thus minimising data-entry errors. Automated data collection methods have been shown to be especially effective in increasing participant willingness to disclose highly-stigmatizing activities or experiences [29]. With participant consent, caregiver interviews are audio-recorded and child assessments are videoed for quality control purposes. Data are checked in weekly batches to allow for constant data-quality monitoring. Tablets are password-protected and data transmitted from them are held on a password-protected central network server.

### Measures

Outcome data are collected through the use of (1) caregiver interviews, (2) direct child assessments, and (3) videoed caregiver-child interactions. Caregiver interviews and child assessments have been translated and back-translated (where applicable) into Sesotho [30]. The primary outcomes are:

1. HIV-testing rate. This is calculated as the number of children who have tested for HIV in the previous 3 months
2. Child language. This is measured using three standardized language measures: the MacArthur Communication Development Inventory [31], a parent-reported measure of early language which was used in the previous study of dialogic book-sharing in Khayelitsha, South Africa [25], the Peabody Picture Vocabulary Test [32], a measure of receptive vocabulary, adapted for use in Lesotho, and the Receptive Language Scale, a subtest of the Mullen Scales of Early Learning [33] which assesses a child’s ability to decode and respond to varying levels of verbal instruction
3. Child attention (Early Childhood Vigilance Task) [34] is measured using a computer-based assessment of sustained attention during which the child is required to watch a screen where various animated stimuli disappear and reappear. This measure was also used in the previous study of dialogic book-sharing in South Africa

Secondary outcomes for child participants include HIV treatment uptake and adherence, cognitive development, executive function, emotional and behavioral functioning, and growth status. For caregivers, secondary outcomes include caregiver mental health and substance use, and parenting outcomes such as discipline, parenting stress, and parental sensitivity. All videoed interactions will be coded by reviewers who are blinded to group allocation. Primary and secondary outcome measures are detailed further in Table 3.

### Data quality control

Individual child assessments and caregiver interviews are videoed and audio-recorded, respectively, and a randomly selected subsample of each is reviewed by a team of data quality controllers. This includes listening to caregiver interviews to ensure that data collectors are not deviating from the prescribed sets of questions and answers, that they are making referrals when appropriate, and that they are treating research participants with respect during all study procedures. Child assessment videos are reviewed to ensure that data collectors are scoring items correctly and following the correct basal and ceiling rules for certain tests. The team receives feedback on an ongoing basis.

### Data analysis plans

Data analysis will be completed by a designated statistician/statistical team that is independent from study investigators. Baseline differences between intervention and control groups will be examined using independent *t* tests [35]. In addition to the primary and secondary outcomes, analysis of baseline differences will also include sociodemographic data such as age, gender, and household poverty.

Intervention efficacy will be evaluated by comparing child outcomes over 12 months between the intervention and control groups. Post-test scores for primary outcomes and secondary outcomes will be used as dependent variables. Parent/child age and gender, allocation group, and baseline scores will be included as covariates [36]. Analyses will also control for intervention site in order to account for variance in implementation among sites [37]. Scores will be compared using multilevel models, (i.e., hierarchical models or random effect models) which can account for clustering of repeated assessments within individuals and clustering of individuals within villages [38, 39]. If data are normally distributed we will fit linear models. If not, we will categorize outcome data and use longitudinal logistic regression to assess differences between intervention and control villages. Intervention effects will be examined using an intention-to-treat analysis [40]. Intention-to-treat considers all participants in an experiment regardless of whether they do or do not complete the program or evaluation [41].

Clustering will be taken into account in all analyses and multiple imputation methods will be used for missing individual outcome data [42]. We will also examine the effect of the intervention dose to assess the relationship between outcomes, phase of intervention, and the number of sessions attended and other process data relating to the implementation of the intervention.

#### Moderator analyses

We will also test potential moderator effects (through subgroup analysis) of whether the intervention works differently for different groups of beneficiaries, such as those living in severe poverty, and grandparents and parents. It will also test effects of caregiver mental health, gender, intervention attendance, fidelity, quality of implementation and other process-related factors.

#### Mediator analyses

Additionally, the study will test potential mediation pathways. For example, whether ECCD training predicts improved child development through increased positive parenting and/or increased healthy stimulation. Analyses will be conducted to examine differences between ECCD attenders and nonattenders whose parents are attending parenting skill training.

Finally, the researchers will seek to cost the intervention and assess its cost-effectiveness subsequent to trial completion.

#### Data sharing

Three years after trial close-out, databases will be made publically available in an accessible format on a relevant public data repository.

#### Power calculation

Power calculations were initially based on two outcome measures that were used in a previous trial of a similar book-sharing methodology in Khayelitsha, the Early Childhood Vigilance Task (ECVT) and the MacArthur Communication Development Inventory (CDI). For this cluster randomized control trial, the sample was calculated to detect a small/medium effect size of 0.3 at 80 % power 12 months post intervention on the CDI, as the more conservative measure. An intracluster correlation (ICC) of 0.05 was used, which is substantially higher than that used in other behavioral studies due to the relative geographical isolation of clusters [39, 43].

First, we calculated the necessary sample size per intervention arm to detect a standardized effect size of 0.30; i.e., a small medium effect size [44] at 12 months with 80 % power. Calculations were carried out in PASS software and assumed a type I error of 0.05 for a two-sided test, two repeated measurements, and an autocorrelation (i.e., the correlation between three adjacent repeated observations) of 0.50 (N* = 123). Second, the sample size per arm was calculated as the product of N* and the variance inflation factor:

$$ N=N* \times \left[1 + \left(m-1\right)\mathrm{I}\mathrm{C}\mathrm{C}\right], $$

where *m* is the average number of children per village (30).

Last, we estimated a loss of 10 % of the sample at each follow-up point. This resulted in our requiring at least 365 children per group in 12 clusters. Due to the small size of some clusters we added additional village clusters to the final sample prior to randomization.

## Discussion

In LMIC, weak health care systems and scarce human resources necessitate that interventions to improve maternal and child health are integrated [21]. Traditionally, vertical programs – often with a narrow disease-focus – have dominated. Increasingly, there has been a focus on horizontally integrated and comprehensive programming for maternal and child health [45]. This is particularly true for early child development and nutrition programs where effectiveness is likely to be markedly increased with the implementation of comprehensive programs [21] and there is a substantial evidence base for this [2, 46]. What is lacking, however, is knowledge of how to combine interventions that target  early child development, nutrition, HIV-testing and retention in care with existing health services [21], particularly in rural populations.

The Sustainable Development Goals (SDG) build on the Millennium Development Goals by stressing sustainable development, not just survival and disease reduction [47], while the Global Strategy for Women’s, Children’s, and Adolescents’ Health proposes a threefold agenda: the Survive (end preventable deaths), Thrive (ensure health and wellbeing), and Transform (expand enabling environments) agenda [48]. The broader developmental focus of the SDG (“leave no one behind”) [49], combined with the thrive agenda of the Global Strategy will require a significant increase in research on integrated programming, beginning in the early years, that evaluates interventions taking place in remote rural areas where significant numbers of the world’s population still live. The goal of the Mphatlalatsane study is to evaluate the impact of an integrated community-based, community-run early childhood care and development intervention that seeks to contribute to national efforts to improve the health and psychosocial wellbeing of vulnerable children aged 5 years and younger in Lesotho. This study provides a unique opportunity to link early child development and nutrition outcome data with HIV-related information for young children in order to improve our knowledge about the nature and extent of the relationship between HIV and child development, and the potential of an integrated early childhood development intervention to prevent or mitigate developmental delays in HIV-infected and -affected children.

The area in which the study is being implemented is a deeply rural site that poses significant geographic barriers. Increasingly, mobile health units are being deployed in Africa in response to deficiencies in health care infrastructure, particularly in remote rural areas in Africa in order to address the service and access gap [50], to serve the needs of remote communities. To date, mobile health units have been widely employed in the realm of HIV care and testing [51] and tuberculosis (TB) testing and referral. However, in very remote areas (such as the Mokhotlong region of Lesotho) adverse weather conditions and impassable roads (during winter) the reach of mobile health units can be compromised. Our integration of mobile health units (health days) with home-based ECCD and nutrition services will, as well as improving the linkages with the primary health care facilities [52], provide essential data on how to deliver comprehensive services to under-served or unserved populations [53].

### Trial status

At the time of manuscript submission participant recruitment was continuing and 75 % complete.

## Abbreviations

ECCD: Early Childhood Care and Development
LMIC: Low- and middle income countries
PEPFAR: President’s Emergency Plan for AIDS Relief
SDG: Sustainable Development Goals
